# Association between triglyceride–glucose index and liver enzymes in adults with metabolic syndrome: a retrospective cross-sectional study

**DOI:** 10.1080/07853890.2025.2522972

**Published:** 2025-06-27

**Authors:** Raju Rana, Shobha U. Kamath, B. Ananthakrishna Shastri, Umakanth Shashikiran, G. Arun Maiya, Ullas Kamath, S. Raghavendra Rao

**Affiliations:** ^a^ Department of Biochemistry, Kasturba Medical College, Manipal, Manipal Academy of Higher Education, Manipal, Karnataka, India; ^b^ Department of Medicine, Kasturba Medical College, Manipal Academy of Higher Education, Manipal, Karnataka, India; ^c^ Department of Medicine, Melaka Manipal Medical College, Manipal Academy of Higher Education, Manipal, Karnataka, India; ^d^ Department of Physiotherapy, Manipal College of Health Professions, Manipal Academy of Higher Education, Manipal, Karnataka, India; ^e^ Department of Biochemistry, Melaka Manipal Medical College, Manipal Academy of Higher Education, Karnataka, India

**Keywords:** Alanine aminotransferase, insulin resistance, metabolic syndrome, non-alcoholic fatty liver disease (NAFLD), triglyceride–glucose index

## Abstract

**Background:**

Previous research has established an association between insulin resistance with alanine aminotransferase (ALT) and aspartate aminotransferase (AST). Studies examining the association between insulin resistance and these enzymes in metabolic syndrome, as determined by the triglyceride–glucose (TyG) index, are scarce. This research aims to investigate the relationship between the TyG index and liver enzyme levels in individuals diagnosed with metabolic syndrome.

**Materials and methods:**

This study was conducted in tertiary care hospitals among the Indian adult population aged 20 to 70 who came for regular health checkups at health checkup units from 2021 to 2022. In total, 12112 data were retrieved from the medical record department. After removing the data that didn’t meet our inclusion criteria, 896 participants with metabolic syndrome, 384 females and 512 males, were enrolled.

**Results:**

Significant differences in AST, ALT, and ALT/AST ratio were observed across TyG index tertiles (*p* < 0.05). The TyG index was a statistically significant positive predictor for (AST, β = 0.56; ALT, β = 1.25, and ALT/AST, β = 0.69, with *p* < 0.001) once correction for age, gender, body mass index, history of diabetes, hypertension, and smoking. Moreover, there was a statistically significant association with several metabolic markers, including total cholesterol, HDL cholesterol, and HbA1C.

**Conclusion:**

The TyG index, a simple and inexpensive metric derived from commonly performed laboratory tests, may be a valuable predictor of abnormal liver enzymes (ALT, AST) and their ratio (ALT/AST) in metabolic syndrome patients.

## Introduction

Metabolic syndrome (MetS) represents a constellation of abdominal obesity, atherogenic dyslipidemia, elevated blood pressure, insulin resistance (IR), and proinflammatory and prothrombotic states that directly amplify the cardiovascular disease (CVD), type 2 diabetes mellitus (T2DM) risk, and all-cause mortality [1]. MetS prevalence has been rising globally, accounting for approximately 41.8% of adult Americans in 2011–12 [2], nearly 30% in South Asian nations [3,4], and 32.4% in African countries [5].

The triglyceride–glucose (TyG) index derived from fasting triglyceride and fasting blood glucose (FBG) is an effective alternative tool to measure IR [6]. It has demonstrated robust associations with standard measures of IR, the homeostasis model assessment of insulin resistance (HOMA-IR), and the hyperinsulinemic–euglycemic clamps technique [7]. The simplicity and accessibility of the TyG index make it an attractive tool for clinical practice and large-scale epidemiological studies to assess IR.

IR represents the central pathophysiological mechanism underlying non-alcoholic fatty liver disease (NAFLD), a significant element for long-term liver damage [8]. NAFLD encompasses two distinct categories: the stable form known as non-alcoholic fatty liver (NAFL) and its more severe counterpart, non-alcoholic steatohepatitis (NASH). While NAFL remains relatively benign, NASH represents a more serious condition that worsens over time. The transformation from NAFL to NASH occurs when liver inflammation and cellular damage emerge alongside existing fat deposits. The features of NASH include excessive fat accumulation in the liver, inflammation, and hepatocyte ballooning with or without perisinusoidal fibrosis. As NASH advances in some patients, it can lead to severe liver damage, ultimately resulting in cirrhosis or liver cancer [9].

Worldwide, NAFLD prevalence is rising, with nearly 32.4% of adults suffering from it [10]. According to a recent meta-analysis, the prevalence of NAFLD among individuals with metabolic disorders (such as MetS, central obesity, diabetes mellitus, and hypertension) was found to be 54.1% in the South Asian Region [11]. According to Chen YF et al. NAFLD is closely associated with increased liver enzymes [12]. IR is a key factor that links MetS and NAFLD. Patients with NAFLD often have one MetS component [13,14]. A comprehensive meta-analysis encompassing 121,975 participants revealed that the odds of developing NAFLD increase with a higher TyG index [15]. Individuals with MetS exhibiting more pronounced IR demonstrate a higher prevalence of advanced fatty liver disease [14].

Even though liver enzymes and the TyG index may both be significant in MetS, the connection between these markers has not been thoroughly studied. Understanding the association in patients with MetS could provide valuable insights into the underlying mechanisms of the condition. Furthermore, the relationship between the TyG index and liver enzymes could provide clinicians with a non-invasive tool to identify early liver dysfunction in MetS patients, improving risk stratification and management strategies. Moreover, exploring this relationship may help elucidate the effects of IR on liver enzymes in MetS.

The present study aims to investigate the association between aspartate aminotransferase (AST), alanine aminotransferase (ALT), the ratio of ALT to AST, and the TyG index. This study’s results could yield significant insights regarding the efficacy of this marker as a non-invasive measure for liver dysfunction within this population.

## Materials and methods

### Study design and setting

In a tertiary care hospital in India, this cross-sectional retrospective study was conducted over two years, from January 1, 2021, to December 30, 2022.

### Study population

**Figure 1.**
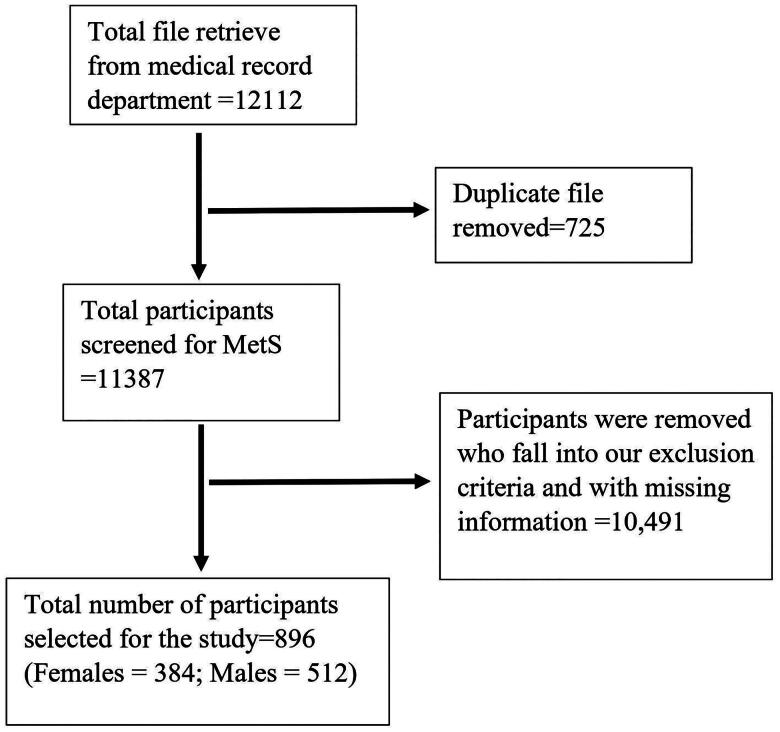
Participant selection flow chart.

Participants were individuals coming to health checkup units of the Kasturba Hospital and Medical College, Manipal, India, for their regular checkups. This study included people aged 20 to 70 who met three criteria of MetS defined by the National Cholesterol Education Program Adult Treatment Panel (NCEP ATP) III [16]. The exclusion criteria were patient data with known cases of cardiovascular disease, cancer, lactation, parathyroid, autoimmune disease, anemia, severe renal disease, a liver disorder like hepatitis, a history of alcohol consumption, and a file with insufficient information. The flow chart below shows the process of selection of participants (Figure 1). This study adhered to the Helsinki Declaration principles. Participants have waived their consent. The Institutional Ethics Committee of Kasturba Medical College and Kasturba Hospital, Manipal, approved the study (Registration number: IEC1: 276/2023) (https://kmckhiec.manipal.edu/) and waived the consent.

## Variables

### Demographic and clinical variables

Patients’ age, blood pressure, height, weight, history of smoking, diabetes, and hypertension were collected from patient’s files.

The formula for calculating body mass index (BMI) was weight in kilograms (kg) divided by height in meters (m) squared.

### Laboratory variables

A blood sample for biochemical parameters was collected after 8–10 h of fasting in the morning. Patients’ FBG, lipid profile, albumin, creatinine, urea, and liver enzymes were measured in Cobas 8000 modular analyzer series (Roche Diagnostics). The high-performance liquid chromatography (HPLC) method was used to determine HbA1C. After two hours of food intake, postprandial blood glucose (PPBG) was measured.

TyG index calculation:

Ln [Triglyceride (mg/dl) × FBS (mg/dL)/2] [6].

**Metabolic syndrome diagnostic criteria (NCEP ATP III**) [16]

Individuals having at least three conditions out of five

1. Waist circumference (Asian cut-off):
  Males: ≥90cm
  Females: ≥80cm
2. Triglycerides: ≥150 mg/dL or on drug treatment.
3. High-density lipoprotein-cholesterol (HDL-C):
  Males: <40 mg/dL
  Females: <50 mg/dL or on medication.
4. High blood pressure:
  Systolic: ≥130 mm Hg
  Diastolic: ≥85 mm Hg or on medication.
5. FBG: ≥100 mg/dL or on medicine for glycemic control.

### Statistical methods

Using the Kolmogorov–Smirnov test, the normality of the continuous variable distribution was evaluated. Normally distributed variables are presented as mean ± standard deviation, while non-normally distributed variables are expressed as median with interquartile range (IQR). For variables with a normal distribution, one-way analysis of variance (ANOVA), and those that were not normally distributed, the Kruskal–Wallis H test was used to compare them among tertiles. Multiple linear regression models were built to look at the relationship between the TyG index and liver enzymes (AST, ALT, and ALT/AST ratio), adjusting for potential confounding factors. Spearman’s rank correlation analyses examined the associations between the TyG index and other relevant variables. A *P* value of less than 0.05 was accepted as statistically significant. The Jamovi (Version 2.3.28) was used to conduct statistical analysis.

## Results

### Sample characteristics

A total of 12112 data were retrieved from the medical record department. After removing the data that didn’t meet our inclusion criteria, we enrolled 896 participants with MetS, which included 384 females and 512 males. The median age for males was 49 (21–70) and for females was 54 (27–70). Out of 896 participants, 320 (35.71%) had a history of diabetes, 396 (44.19%) with history of hypertension, and 44 (4.91%) were smokers.

### Variables across TyG index tertiles

Comparative analysis among different tertile of the TyG index shows significant differences among AST, ALT, the ratio of ALT to AST (*p* < 0.05), and other biochemical parameters with a rise in the TyG index, as shown in Table 1. Further, BMI was statistically significantly high with lower TyG index values.

**Table 1.** Comparison of variables among TyG index tertiles.

Variables	T1 (*n* = 294)	T2 (*n* = 295)	T3 (*n* = 307)	*P*
Sex				
Male *n* (%)	129 (43.87)	183 (62.03)	200 (65.15)	–
Female *n* (%)	165 (56.12)	112 (37.96)	107 (34.85)	–
Age (years)	52 (21–70)	51 (22–69)	52 (23–70)	0.968
Systolic blood pressure (mmHg)	140 [26]	140 [30]	138 [30]	0.491
Diastolic blood pressure (mmHg)	90 [10]	86 [10]	84 [10]	0.180
Body mass index (kg/m^2^)	28.4 [6.20]	27.6 [5.05]	26.9 [5.03]	<0.001
Fasting blood glucose(mg/dL)	105 [14]	111 [24]	155 [88]	<0.001
Triglyceride (mg/dL)	135 [57.5]	191 [47.5]	229 [117]	<0.001
HDL-C (mg/dL)	39 [10]	36 [9]	36 [9]	<0.001
HbA1C (%)	5.9 [0.80]	6.1 [1.20]	8.1 [3.25]	<0.001
Postprandial blood glucose (mg/dL)	121 [56.5]	139 [75]	230 [149]	<0.001
Urea (mg/dL)	20 [8]	20 [8]	19 [9]	0.600
Creatinine (mg/dL)	0.80 [0.24]	0.85 [0.26]	0.83 [0.25]	0.003
Albumin (mg/dL)	4.56 [0.31]	4.60 [0.30]	4.60 [0.40]	0.020
ALT (IU/L)	23 [16]	26[21]	30 [23.5]	<0.001
AST (IU/L)	21 [9]	22[9.5]	23 [11]	0.006
ALT/AST	1.09 [0.45]	1.16[0.53]	1.25 [0.49]	<0.001
ALP (U/L)	78 [24.8]	78[27.5]	84 [27.5]	<0.001
TyG index	8.92 [0.33]	9.30[0.19]	9.81 [0.46]	<0.001
LDL-C (mg/dL)	129 ± 36.4	137 ± 38.7	135 ± 38	0.046
Total cholesterol (mg/dL)	187 ± 39.8	199 ± 42.5	206 ± 42.5	<0.001
Total cholesterol/HDL-C	4.86 ± 1.20	5.37 ± 1.21	5.60 ± 1.37	<0.001

*Note.* Variables are shown in mean ± SD, median [interquartile range], number (percentage), and age in range.

T: tertile; ALT: alanine aminotransferase; HDL-C: high-density lipoprotein cholesterol; ALP: alkaline phosphatase; AST: aspartate aminotransferase; LDL-C: low-density lipoprotein cholesterol.

### Linear regression analysis

Linear regression models were applied to investigate the relationship between liver enzyme (AST, ALT, and ALT/AST ratio) as dependent variables and the independent variable, the TyG index, as shown in Table 2. The TyG index was a positive predictor for (AST, β = 0.56; ALT, β = 1.25, and ALT/AST, β = 0.69, *p* < 0.001 at 95% CI) adjusted for confounding factors. After controlling for variables, the significance of this association reduces.

**Table 2.** Linear regression analysis for liver enzymes.

	Aspartate aminotransferase (AST)				
SN	β	SE	95% CI		***P***
			Lower	Upper	
TyG index					
Crude	0.675	0.234	0.03	0.16	0.004
Model 1	0.39	0.233	−0.009	0.121	<0.001
Model 2	0.56	0.25	0.009	0.15	<0.001
	Alanine aminotransferase (ALT)				
	β	SE	95% CI		*P*
			Lower	Upper	
TyG index					
Crude	1.79	0.33	0.11	0.24	<0.001
Model 1	1.19	0.32	0.06	0.18	<0.001
Model 2	1.25	0.34	0.06	0.19	<0.001
	ALT/AST				
	β	SE	95% CI		*P*
			Lower	Upper	
TyG index					
Crude	1.12	0.19	0.12	0.25	<0.001
Model 1	0.80	0.19	0.07	0.19	<0.001
Model 2	0.69	0.20	0.05	0.18	<0.001

Model 1: corrected for age and gender.

Model 2: further corrected for BMI, history of smoking, diabetes, and hypertension.

All variables are log-transformed, CI: confidence interval.

### Correlation of TyG index with various biochemical and clinical parameters

Table 3 presents the correlation analysis results. The TyG index demonstrated a weak inverse association with HDL-C levels. Conversely, it positively correlated with total cholesterol (TC) and the TC to HDL-C ratio. These associations were all statistically significant (*p* < 0.001). A statistically significant but moderate association was detected with PPBS (*r* = 0.520, *p* < 0.001) and HbA1C (*r* = 0.464, *p* < 0.001). Further, A small but significant inverse relationship with BMI and a positive with liver enzymes was found (*p* < 0.05).

**Table 3.** TyG Index correlation with other variables.

SN	TyG index	
	*R*	*P*
Systolic blood pressure (mmHg)	−0.034	0.307
Diastolic blood pressure (mmHg)	−0.043	0.197
Body mass index (kg/m^2^)	−0.155	<0.001
Total cholesterol (mg/dL)	0.217	<0.001
HDL-C (mg/dL)	−0.192	<0.001
HbA1c (%)	0.464	<0.001
Postprandial blood glucose (mg/dL)	0.520	<0.001
Urea (mg/dL)	−0.028	0.397
Albumin (mg/dL)	0.119	<0.001
Creatinine (mg/dL)	0.031	0.358
Alanine aminotransferase (ALT) (IU/L)	0.184	<0.001
Alkaline phosphatase (U/L)	0.143	<0.001
Aspartate aminotransferase (AST) (IU/L)	0.106	0.002
ALT/AST	0.182	<0.001
LDL-C (mg/dL)	0.069	0.040
Total cholesterol/HDL-C	0.309	<0.001

HDL-C: high-density lipoprotein cholesterol; LDL-C: low-density lipoprotein cholesterol.

## Discussion

This investigation examines the interplay between hepatic enzyme levels and TyG index in individuals with MetS. Regression analyses found it to predict both ALT and AST levels significantly. Furthermore, a notable correlation was found between the ALT/AST ratio and the TyG index. Importantly, these associations remained robust after correcting for confounding variables, underscoring a compelling link between this index and hepatic dysfunction in the context of MetS. Significant differences in hepatic enzyme levels and the ratio of ALT to AST were observed across the TyG index tertiles. These findings suggest a robust association between IR and hepatic function.

This finding is consistent with previous research demonstrating a relationship between this index and liver disorders [17–19]. Bonnet et al. [20] demonstrated a substantial association between ALT activity and IR in both peripheral tissues and the liver, as well as diminished hepatic insulin extraction, among healthy individuals of both sexes, which shows similar trends to our study. The progressive increase in liver enzymes with higher TyG index tertiles may indicate a dose–response relationship, further strengthening the applicability of this index as a surrogate measure of liver dysfunction in the context of IR. Furthermore, in a cohort of MetS patients, this index was identified as a predictor of non-alcoholic steatohepatitis (NASH) [21].

The results of this investigation were in accordance with a study conducted on normal-weight Japanese adults. In this study, IR was positively correlated with ALT to AST ratio [22]. Similarly, this relationship was seen in both centrally obese and non-obese Chinese populations. In this study, IR was measured by HOMA-IR [23].

A complex pathophysiological mechanism may underpin the observed association between IR and elevated hepatic enzymes. Central to this process is the impaired glucose uptake in insulin-resistant tissues. This impairment could result in excessive glucose being reabsorbed into the liver, inhibiting gluconeogenesis and promoting lipogenesis. This process might lead to hepatic fat accumulation and inflammation, potentially compromising liver structure and elevated liver enzymes. Further, studies have demonstrated a link between NAFLD, a hepatic manifestation of MetS, and chronic elevation of liver enzymes, particularly ALT [22,24]. Furthermore, it was shown that the quantity of fat deposited in the liver is linked to the ALT to AST ratio value [25,26].

The robust correlation between the TyG index and liver enzymes suggests it could be an affordable screening tool for MetS patients at high risk of liver impairment. This marker could serve as important clinical implications, such as early detection of liver abnormalities, primary care screening of asymptomatic patients with metabolic risk factors, initial risk assessment, evaluation of individuals with a family history, and assessment of patients with normal liver enzyme levels but significant metabolic abnormalities. Integration with conventional liver enzyme assessments may enhance risk stratification accuracy. Eventually, this could lead to timely interventions and potentially prevent the progression to more severe liver disease. Our findings could help establish early predictive markers for NAFLD in this population. Moreover, South Asia faces a particularly high burden of both NAFLD (33.8%) and NASH (5.42%), highlighting the urgent need for cost-effective early screening markers in this region [27]. Future prospective studies should focus on establishing population-specific optimal cut-off values, particularly in MetS patients.

This investigation possesses several notable strengths that enhance the validity and applicability of its findings. A primary strength of this investigation lies in its utilization of a larger population size, which significantly enhances the findings’ statistical robustness and external validity. Utilizing the TyG index for liver dysfunction evaluation is a key methodological advantage. This index offers a cost-effective alternative to more complex measures, as it is derived from routinely assessed clinical parameters triglycerides and fasting blood glucose. The accessibility and simplicity of the TyG index make it particularly valuable for large-scale epidemiological studies and clinical practice in resource-limited settings. In settings where imaging techniques (such as ultrasound or MRI) or advanced biomarkers are unavailable, the TyG index serves as an accessible, cost-effective screening tool to identify individuals who may require further evaluation.

However, some limitations should be acknowledged. The cross-sectional design precludes the assessment of temporal trends and causal relationships. Longitudinal studies are needed to validate these findings and elucidate potential causative pathways. The absence of liver imaging data limited our ability to confirm NAFLD diagnosis; future research should incorporate advanced imaging techniques alongside this marker for comprehensive validation. Potential unmeasured confounding factors, such as diet, physical activity, and alcohol use, may have influenced liver enzyme levels. Moreover, we were unable to compare the TyG index with other validated measures of insulin resistance. Future prospective studies should evaluate the relative performance of the TyG index against conventional insulin resistance metrics to establish its comparative utility and clinical applicability in this population.

## Conclusion

In conclusion, our results show that in patients with MetS, there is a strong correlation between the TyG index and liver enzymes. This relationship highlights the interconnected nature of IR, lipid metabolism, and liver dysfunction in MetS. This marker could be a valuable tool in this population to identify those at high risk of liver abnormalities, allowing for earlier management and intervention approaches.

Future research should concentrate on prospective studies to ascertain this marker’s predictive utility for advancing liver disease. Its accessibility and cost-effectiveness make it a promising tool for widespread screening and preventive healthcare initiatives.

## Consent for publication

Not applicable.

## Data Availability

The datasets used and analyzed during this study are available from the corresponding author upon reasonable request.
